# Quantitative Magnetic Resonance Imaging Biomarkers in the Evaluation of Disease Activity in Thyroid Eye Disease: A Retrospective Study

**DOI:** 10.7759/cureus.101075

**Published:** 2026-01-08

**Authors:** Duncan Marston, Sarah Grech, Andrea Noah Paris, Nicole Galdes, Isaac Bertuello, Andrew Palmier

**Affiliations:** 1 Ophthalmology, Mater Dei Hospital, Msida, MLT; 2 Surgery, Mater Dei Hospital, Msida, MLT

**Keywords:** extraocular muscles, mri orbit, neuroimaging biomarkers, orbital imaging, ‘thyroid eye disease’

## Abstract

Objective

The aim of this study was to determine whether quantitative measurements of extra-ocular muscles and structures on magnetic resonance imaging (MRI) correlate with disease activity in thyroid eye disease (TED).

Method

A retrospective review of 162 patients, between June 2014 and June 2024, with TED who underwent MRI orbits was conducted. The patients were categorised into two groups: those with clinical and imaging features consistent with active TED (n = 73; 45.1%) and those with inactive disease (n = 89; 54.9%). T1-weighted imaging, T2-weighted imaging, and short tau inversion recovery (STIR) on MRI sequences were used to measure exophthalmos value, extra-ocular muscle diameters, orbital fat thickness, and optic nerve diameters. An independent t-test was carried out to determine statistical significance (p < 0.05) in structural measurements between both groups. The area under the receiver operating characteristic curve (ROC) analysis was used to deduce the diagnostic accuracy of structural measurements in predicting disease activity.

Results

Inferior rectus (IR) and medial rectus (MR) muscles were significantly larger in patients with active disease (p < 0.05 and p < 0.05, respectively). Total extraocular muscle (EOM) was also significantly larger in this group (p < 0.05). Total EOM/exophthalmos and IR/total EOM ratios showed higher values in patients with active disease (p < 0.05). ROC analysis showed that the cut-off point of total EOM/exophthalmos ratio was 0.647, with a sensitivity of 90.4% and specificity of 88.8% (AUC = 0.96; 95% CI: 0.94-0.99), whilst the IR/Total EOM ratio had a cut-off point of 0.362, with a sensitivity of 90.4% and a specificity of 87.6% (AUC = 0.94; 95% CI: 0.9-0.98).

Conclusion

MRI-derived quantitative measurements, particularly the total EOM/exophthalmos and IR/total EOM ratios, reliably distinguish active from inactive TED, on MRI, with high sensitivity and specificity. These imaging biomarkers offer a valuable, non-invasive tool for assessing disease activity and monitoring progression in TED.

## Introduction

Thyroid eye disease (TED), also referred to as Graves’ ophthalmopathy, is an autoimmune inflammatory condition that predominantly affects the orbital and periorbital structures, encompassing the extraocular muscles (EOM) and orbital adipose tissue [1]. It constitutes the most prevalent cause of orbital disorders in adults, occurring in approximately 25-50% of individuals with Graves’ disease (GD), an autoimmune thyroid condition characterized by thyroid-stimulating immunoglobulins (TSI) and thyrotropin receptor antibodies (TRAb) [2,3]. TED typically presents with varying degrees of proptosis, eyelid retraction, diplopia, and, in severe instances, compressive optic neuropathy, all of which can considerably hinder visual performance and quality of life [4].

The natural trajectory of TED was initially described by Rundle, who characterized its progression through a triphasic curve consisting of an initial active inflammatory phase (often termed as the “wet phase”), followed by a fibrotic or regression phase, and ultimately reaching a quiescent phase (or “dry phase”) [5]. Accurate recognition of disease activity is vital for clinical decision-making, as active inflammatory disease frequently responds favorably to immunosuppressive or radiotherapeutic treatments, while inactive fibrotic disease is more suitable for surgical intervention [6,7]. However, distinguishing between these phases through clinical signs alone can be difficult.

The Clinical Activity Score (CAS), which is based on indicators such as pain, redness, and swelling, remains the most commonly employed clinical instrument for evaluating disease activity [8]. Nonetheless, CAS is subjective and may not entirely encapsulate the underlying orbital inflammatory load. Imaging techniques, particularly magnetic resonance imaging (MRI), provide an objective, non-invasive supplement for assessing both the morphological and inflammatory elements of TED [9,10]. MRI offers superb soft-tissue contrast, and specific sequences such as short tau inversion recovery (STIR) and T2-weighted imaging can identify increased signal intensity linked to heightened water content and active inflammation, while T1-weighted imaging aids in the evaluation of fat proliferation and structural alterations [11-13].

Despite its diagnostic capabilities, there is still no agreement regarding the most dependable quantitative MRI biomarkers to evaluate TED activity. Previous investigations have demonstrated that enlargement of the EOM and expansion of orbital fat correlate with disease activity; however, most have depended on qualitative evaluations or limited patient groups [14-15]. Therefore, the creation of reproducible, quantitative MRI metrics could enhance diagnostic precision and facilitate improved monitoring of the disease.

Consequently, the primary aim of this study was to ascertain whether quantitative MRI-derived parameters, namely, EOM diameters, exophthalmos, orbital fat thickness, and optic nerve diameter, are associated with TED activity. A secondary aim was to evaluate the diagnostic accuracy of derived ratios utilizing receiver operating characteristic (ROC) analysis to differentiate between active and inactive disease. By establishing reliable imaging biomarkers, this study seeks to provide clinicians with objective and reproducible tools for assessing disease activity and guiding personalized management in patients with TED.

## Materials and methods

Study design and population

This was a retrospective, descriptive, and comparative study conducted at Mater Dei Hospital, Malta. The subjects in this study were recruited during a retrospective assessment of MRI orbits performed for patients with confirmed TED between June 2014 and June 2024 at Mater Dei Hospital, Malta. A total of 162 patients were enrolled in the study after screening for inclusion and exclusion criteria. 

The inclusion criteria were as follows: (1) Patients had to be 18 years or older to participate, (2) have a confirmed diagnosis of Graves’ disease as per the European Thyroid Association guideline for the management of Graves’ hyperthyroidism, and (3) have a recorded CAS as per the European Group on Graves’ Orbitopathy (EUGOGO) guidelines. Exclusion criteria were as follows: (1) history of orbital surgery, (2) cases with monocular involvement, (3) known orbital or ophthalmic compressive pathology, (4) patients who did not complete the MR study, (5) history of orbital irradiation, and (6) systemic disease with possible orbital involvement other than thyroid disease.

After evaluating the CAS, patients were classified into two groups: active (CAS > 3) and inactive (CAS < 3) TED. Orbital MRI scans were then analyzed to support this classification. T2-weighted images (T2WI) and STIR sequence images were used to determine inflammation in the extra-ocular muscles [6,7], whilst T1-weighted images (T1WI) were used to determine inflammation in the orbital fat [8].

MRI acquisition

MRI scans were performed using a Signa Lift 1.5T MR scanner (GE HealthCare, Chicago, Illinois, USA) with patients in the supine position. Orbital images were acquired in transverse, coronal, and oblique sagittal planes. The T1-weighted coronal images were obtained with a slice thickness of 3 mm, acquisition time of 3.49 ms, pixel size of 0.6 × 0.7 mm, repetition time (TR) of 440 ms, echo time (TE) of 12 ms, and a field of view (FOV) of 180 × 180 mm. T1-weighted axial images were acquired with a slice thickness of 3 mm, acquisition time of 3.51 ms, pixel size of 0.4 × 0.6 mm, TR of 3720 ms, TE of 68 ms, and FOV of 180 × 180 mm. Coronal T2-weighted and STIR sequences were obtained with a slice thickness of 3 mm, acquisition time of 4.55 ms, pixel size of 0.6 × 0.8 mm, TR of 2540 ms, TE of 30 ms, and FOV of 180 × 180 mm.

Measurement of ophthalmic parameters

**Figure 1:**
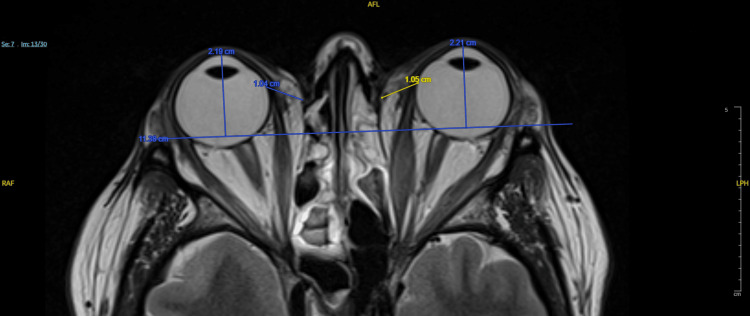
Measurement of exophthalmos (mm) and orbital fat thickness (mm) on axial T2WI T2WI: T2-weighted image

**Figure 2:**
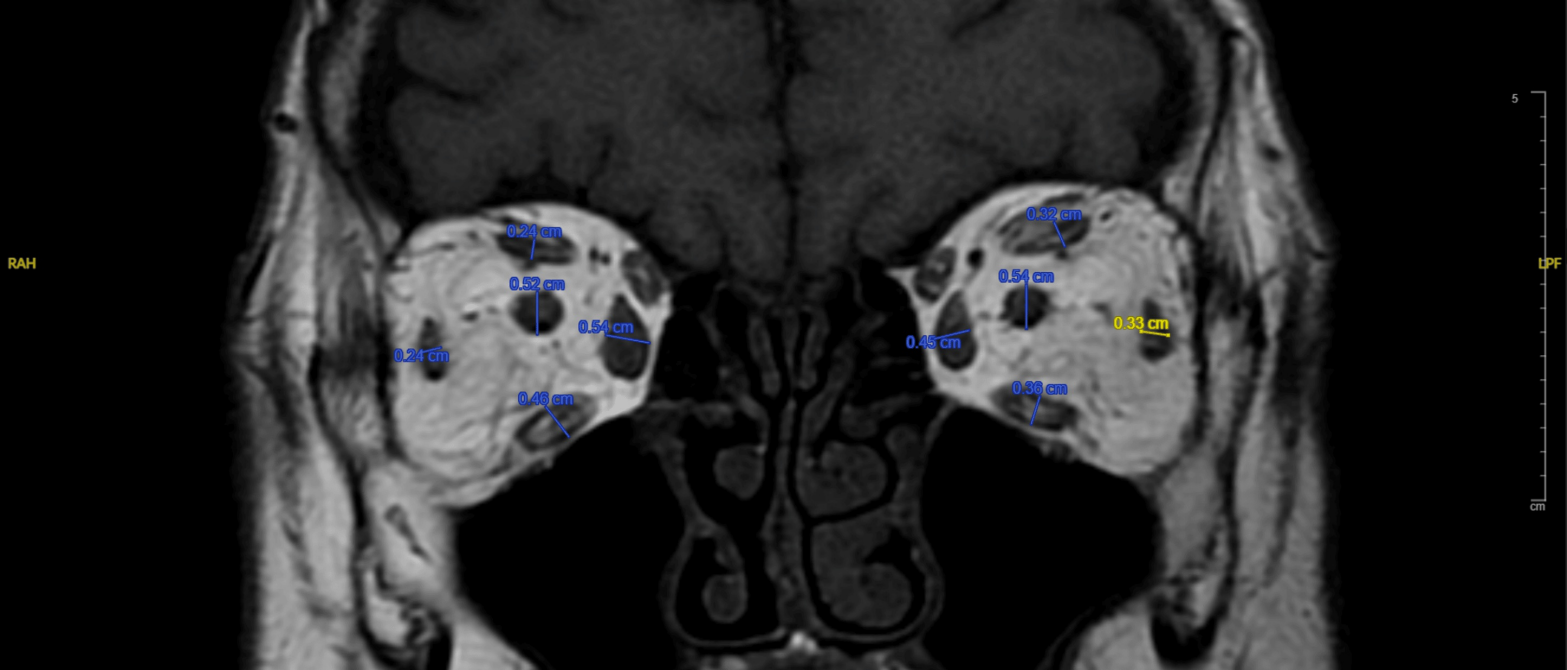
Measurement of extraocular muscles (mm) and optic nerve diameter (mm) on coronal T1WI T1WI: T1-weighted image

Ophthalmic parameters were measured as follows. Exophthalmos was determined on T2-weighted cross-sectional MR images by selecting the slice showing the maximal display of the eyeball and orbital structures; a horizontal line was drawn between the bilateral zygomatic arches, and the vertical distance from this line to the anterior corneal surface was recorded as the exophthalmos value (mm) (Figure 1). EOM thickness was measured on T1-weighted coronal images, with the horizontal diameter used for the medial and lateral rectus muscles and the vertical diameter for the superior and IR muscles. Successive measurements were performed for each muscle, and the largest diameter was recorded, with the total EOM thickness calculated as the sum of all four muscles; the superior and inferior oblique muscles were not measured (Figure 2). Orbital fat thickness was measured on T2-weighted cross-sectional images as the maximal distance from the lateral border of the MR muscle at the globe to the medial orbital wall (mm) (Figure 1). Optic nerve diameter was measured on coronal T1WI as the maximal nerve diameter (mm) (Figure 2).

To minimise inter-observer variability, all measurements were conducted by two clinicians who followed pre-defined anatomical landmarks and adhered to step-by-step guidelines to ensure consistency and accuracy. The observers were kept unaware of the clinical activity status, and measurements were taken independently, with any discrepancies resolved through consensus discussion. Consistent imaging sequences, slice thickness, and plane orientation were used. Furthermore, prior to starting data collection, the involved clinicians carried out an assessment of pilot scans to verify consistency. Collectively, these measures reduced observer-related variability, ensuring that the MRI-derived parameters offered a solid and trustworthy foundation for assessing disease activity in TED.

Statistical analysis

Statistical analysis was carried out using IBM SPSS Statistics for Windows, Version 29 (Released 2022; IBM Corp., Armonk, New York, United States). For every individual, the average of both eyes was taken for each ophthalmic parameter. We presented the mean, standard deviation, effect size, p-value, and confidence interval for each parameter. The differences between the active and inactive TED groups were analysed using the independent t-test. The Pearson correlation test was used to assess for linear correlation between the ophthalmic parameters for both groups. ROC curve analysis was used to assess the degree of sensitivity and specificity of various ophthalmic parameters to help predict disease activity on MRI. A p-value < 0.05 was considered statistically significant.

Ethics

The study was conducted in accordance with ethical principles derived from the Declaration of Helsinki and adhered to Good Clinical Practice guidelines. The study protocol received approval from the ethics committee of Mater Dei Hospital and the chairman of the ophthalmology department.

## Results

Clinical and imaging characteristics

In our retrospective study, we managed to collect the MRIs of 162 patients over a ten-year period. Active TED was documented in 45.1% (n = 73) of the patients, whilst 54.9% (n = 89) of the patients had inactive TED. A higher proportion of female patients was noted in both groups, 55 females in the active group and 67 females in the inactive group. The average age of the patients in the active group was 54.1 ± 14.78, whilst in the inactive group, it was 55.81 ± 14.52. Thyroid function tests and associated immunological markers were collected and are illustrated in Table 1. In the active TED group, 20.5% (n = 15) of patients were active smokers compared to 10.1% (n = 9) of patients in the inactive TED group. 

**Table 1:** Comparison of thyroid function tests and TSH receptor antibody levels between active and inactive TED TSH: thyroid-stimulating hormone; TED: thyroid eye disease Data are presented as mean ± standard deviation (SD), with standard error (SE) in parentheses. p-values represent comparisons between active and inactive TED groups. A p-value < 0.05 was considered statistically significant

Variable	Active TED (n = 73), mean ± SD (SE)	Inactive TED (n = 89), mean ± SD (SE)	p-value
Serum T4 (11.9-20.3 pmol/L)	19.42 ± 11.34 (1.35)	15.39 ± 3.92 (0.44)	<0.05
Serum T3 (3.5-6.5 pmol/L)	8.21 ± 6.43 (1.31)	5.72 ± 1.76 (0.30)	<0.05
TSH level (0.3-3 micIU/ml)	3.63 ± 6.20 (0.95)	1.88 ± 2.16 (0.25)	<0.05
TSH receptor antibody (0.1-1 IU/L)	11.54 ± 11.73 (1.68)	6.09 ± 7.24 (1.27)	<0.05

Table 2 summarizes the treatment history of patients with active and inactive TED.

**Table 2:** Treatment history of patients with active and inactive TED TED: thyroid eye disease Values are presented as the number of patients (percentage). Patients may have received more than one treatment modality

Treatment	Active TED (n = 73), n (%)	Inactive TED (n = 89), n (%)
Carbimazole	24 (32.9%)	27 (30.3%)
Thyroid surgery	11 (15.1%)	12 (13.5%)
Steroids	13 (17.8%)	5 (5.6%)
Selenium	7 (9.6%)	3 (3.4%)
Azathioprine	0 (0%)	1 (1.1%)
Radioactive iodine	0 (0%)	3 (3.4%)
No active treatment	22 (30.1%)	21 (23.6%)

Table 3 provides a comparison of the orbital parameters measured between the active and inactive TED groups.

**Table 3:** Comparison of orbital imaging parameters between active and inactive thyroid eye disease EOM: extraocular muscle

	Active disease	Inactive disease	Effect size	p-value	95% CI
Exophthalmos (mm)	21.02 ± 2.83	21.83 ± 3.23	0.77	0.09	(-0.18,1.73)
						Orbital fat thickness (mm)	9.03 ± 1.46	9.04 ± 1.86	0.006	0.97	(-0.52,0.53)
Optic nerve diameter (mm)	4.99 ± 0.78	4.99 ± 0.82	0.007	0.98	(-0.24,0.26)
Total EOM thickness (mm)	17.07 ± 3.70	15.62 ± 3.00	-1.44	<0.05*	(-2.49, -0.41)
Lateral rectus (mm)	3.38 ± 1.03	3.29 ± 1.02	-0.1	0.52	(-0.42,0.21)
Inferior rectus (mm)	5.38 ± 1.35	4.79 ± 1.06	-0.58	<0.05*	(-0.96, -0.21)
Medial rectus (mm)	4.91 ± 1.43	4.35 ± 1.05	-0.55	<0.05*	(-0.94, -0.17)
Superior rectus (mm)	3.39±1.07	3.17±1.03	-0.22	0.194	(-0.22,0.17)

Table 4 provides an illustrated presentation of orbital parameter ratios between the active and inactive TED groups. 

**Table 4:** Ratio-based comparison of orbital parameters between active and inactive thyroid eye disease EOM: extraocular muscle; SD: standard deviation; CI: confidence interval; IR: inferior rectus Values are mean ± SD. Mean difference presented as active-inactive with 95% CI. Total EOM thickness is the sum of four rectus muscle maximal diameters in millimetres. Two-sided tests; p < 0.05. Exploratory comparisons; primary endpoints are total EOM/exophthalmos and IR/total EOM ratios

	Active disease	Inactive disease	Effect size	p-value	95% CI
Total EOM/exophthalmos	0.82 ± 0.17	0.52 ± 0.09	-0.29	<0.05*	(-0.34, -0.26)
Inferior rectus/exophthalmos	0.26 ± 0.06	0.22 ± 0.04	-0.04	<0.05*	(-0.05, -0.02)
Inferior rectus/orbital fat thickness	0.61 ± 0.18	0.55 ± 0.19	-0.06	0.05	(-0.11, -0.001)
Orbital fat thickness/exophthalmos	0.43 ± 0.06	0.42 ± 0.07	-0.02	0.13	(-0.04, 0.01)
Orbital fat thickness/total EOM	0.55 ± 0.12	0.82 ± 0.19	0.28	<0.05*	(0.23, 0.33)
Inferior rectus/total EOM	0.31 ± 0.04	0.43 ± 0.06	0.11	<0.05*	(0.1, 0.13)

Pearson’s correlation

Pearson correlation analysis was performed to assess the relationships between MRI-derived parameters in both the active and inactive TED groups. In the active group, there was a significant positive correlation between orbital fat thickness and exophthalmos (r = 0.55, p < 0.001), as well as between total EOM volume and exophthalmos (r = 0.344, p = 0.003). A weak negative correlation was observed between optic nerve diameter and orbital fat thickness (r = -0.23, p = 0.049), whilst no significant association was found between optic nerve diameter and total EOM volume (r = -0.121, p = 0.31). Similarly, in the inactive group, orbital fat thickness showed a strong positive correlation with exophthalmos (r = 0.58, p < 0.001), and total EOM volume was moderately correlated with exophthalmos (r = 0.50, p < 0.001). A weak negative correlation was detected between optic nerve diameter and total EOM volume (r = -0.21, p = 0.049), whereas no significant correlation was found between optic nerve diameter and orbital fat thickness (r = -0.06, p = 0.61).

ROC curve analysis

**Figure 3:**
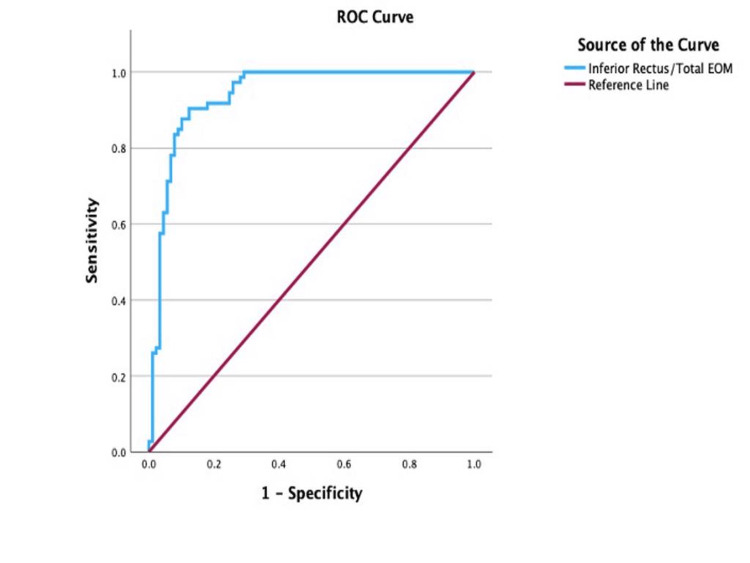
Receiver operating characteristic (ROC) curve for the inferior rectus/total EOM ratio in differentiating active from inactive thyroid eye disease EOM: extraocular muscle

An ROC curve analysis of ocular imaging parameters was carried out to determine their sensitivity and specificity in predicting disease activity at cut-off points. The ROC analysis of the IR/total EOM, illustrated in Figure 3, reported a cut-off point for disease activity to be at 0.362 (AUC = 0.94; 95% CI: 0.9-0.98; sensitivity, 90.4%; specificity, 87.6%; p < 0.001). With values equal to or greater than 0.362 indicative of inactive disease.

**Figure 4:**
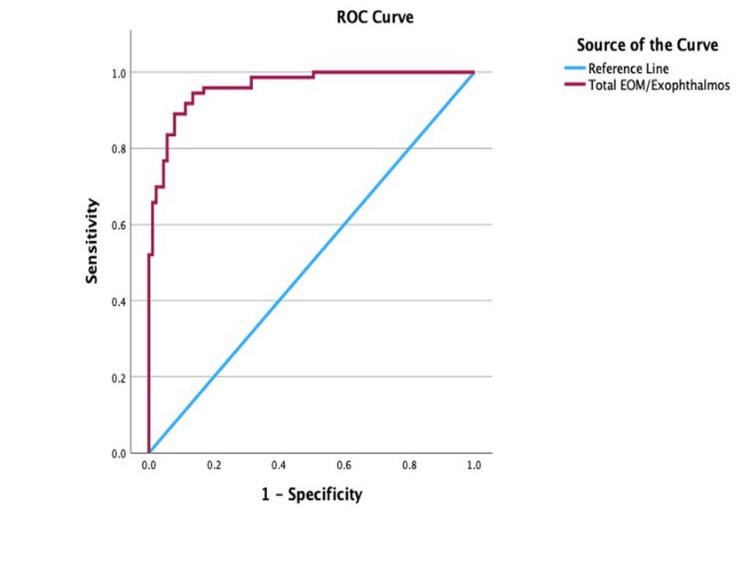
Receiver operating characteristic (ROC) curve for the total EOM/exophthalmos in determining disease activity EOM: extraocular muscle

ROC analysis of the total EOM/exophthalmos, illustrated in Figure 4, reported a cut-off point for disease activity to be at 0.647 (AUC = 0.96; 95% CI: 0.94-0.99; sensitivity, 90.4%; specificity, 88.8%; p < 0.001), with values equal to or greater than 0.647 indicative of active disease.

**Figure 5:**
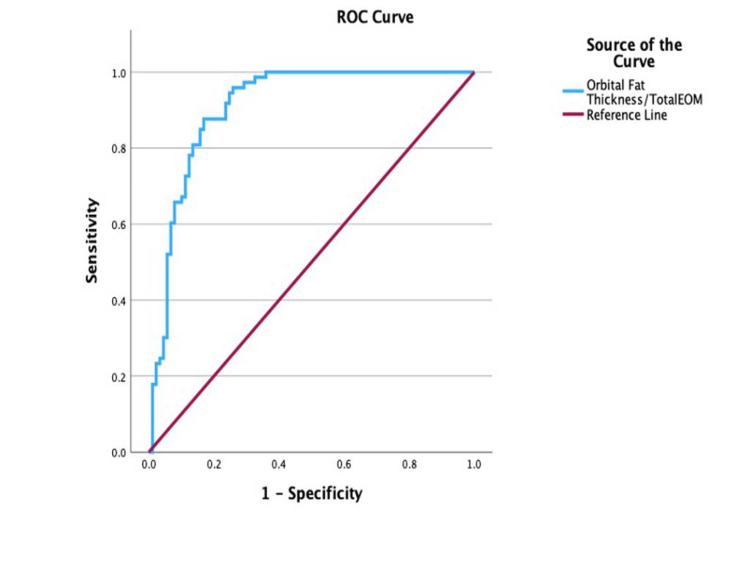
Receiver operating characteristic (ROC) curve for the orbital fat thickness/total EOM ratio in determining disease activity EOM: extraocular muscle

ROC analysis of the orbital fat thickness/total EOM, illustrated in Figure 5, reported a cut-off point for disease activity to be at 0.66 (AUC = 0.91; 95% CI: 0.86-0.96; sensitivity, 87.7%; specificity, 83.1%; p < 0.001), with values equal to or greater than 0.66 indicative of inactive disease.

**Figure 6:**
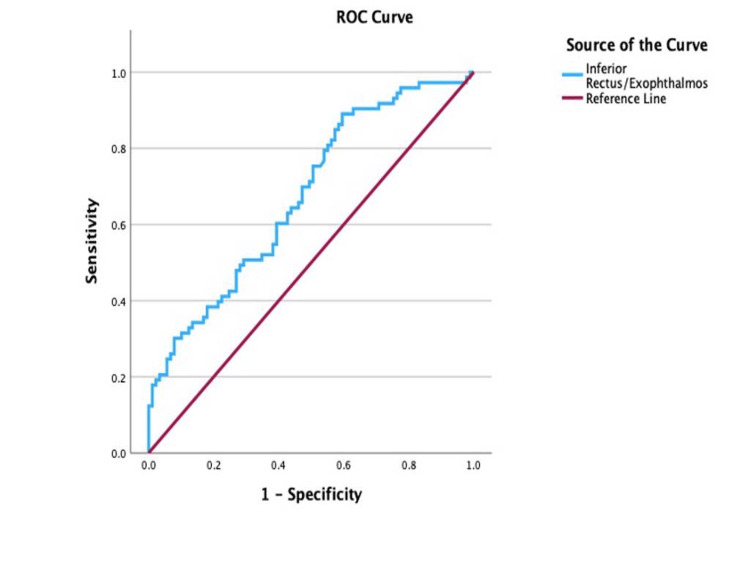
Receiver operating characteristic (ROC) curve for the inferior rectus/exophthalmos ratio in determining disease activity

ROC analysis of the IR/exophthalmos, illustrated in Figure 6, reported a cut-off point of 0.22 (AUC = 0.68; 95% CI: 0.59-0.76; sensitivity, 75.3%; specificity, 49.4%; p < 0.001), with values equal to or greater than 0.22 indicative of active disease.

**Figure 7:**
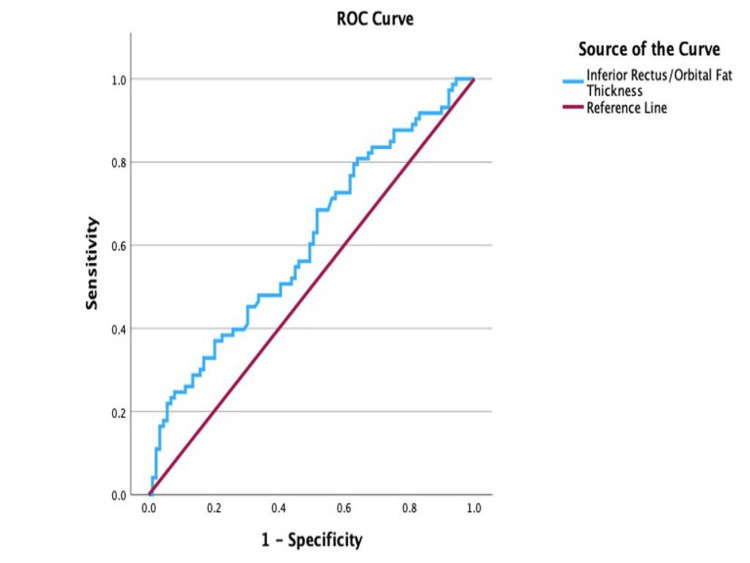
Receiver operating characteristic (ROC) curve for the inferior rectus/orbital fat thickness ratio in determining disease activity

ROC analysis of the IR/orbital fat thickness, shown in Figure 7, reported a cut-off point at 0.61 (AUC = 0.61; 95% CI: 0.52-0.69; sensitivity, 68.5%; specificity, 48.5%; p = 0.02), with values equal to or greater than 0.61 indicative of active disease.

**Figure 8:**
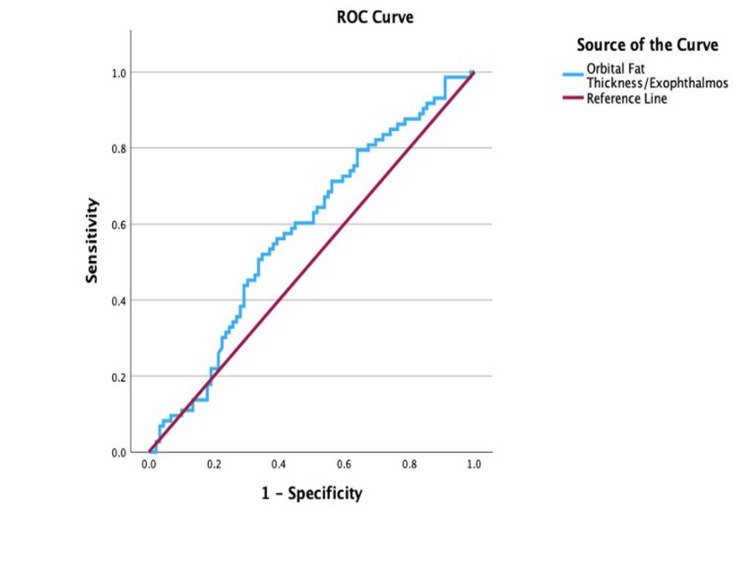
Receiver operating characteristic (ROC) curve for the orbital fat thickness/exophthalmos ratio in determining disease activity

ROC analysis of the orbital fat thickness/exophthalmos, illustrated in Figure 8, reported a cut-off point of 0.43 (AUC = 0.58; 95% CI: 0.49-0.67; sensitivity, 60.3%; specificity, 55.1%; p = 0.08), with values equal to or greater than 0.43 indicative of active disease.

**Figure 9:**
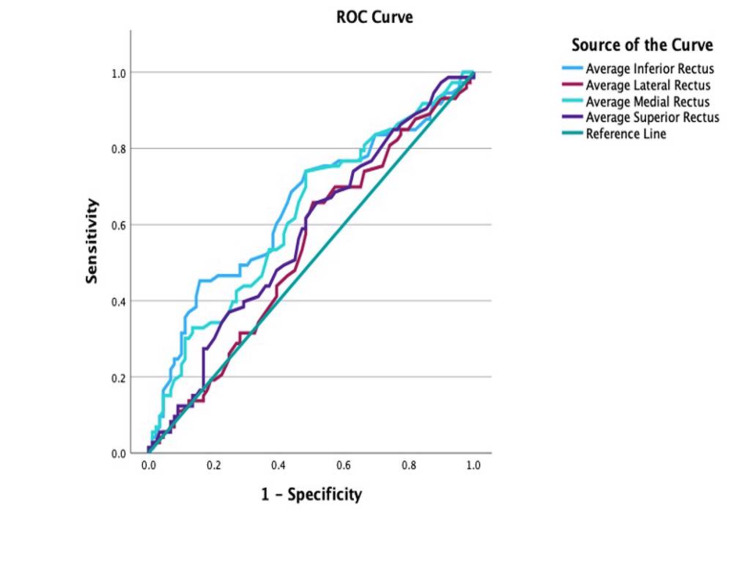
Receiver operating characteristic (ROC) curve for the individual extraocular muscles in determining disease activity

ROC analysis of the IR and MR, both illustrated in Figure 9, reported a cut-off point for the IR at 4.68 (AUC = 0.65; 95% CI: 0.56-0.73; sensitivity, 74%; specificity, 51.7%; p = 0.001), with values equal to or greater than 4.78 indicative of active disease. A cut-off point for the MR muscle for disease activity was calculated to be at 4.13 (AUC = 0.62; 95% CI: 0.54-0.71; sensitivity, 74%; specificity, 51.7%; p = 0.005), with values equal to or greater than 4.13 indicative of active disease.

## Discussion

MRI is a well-established imaging modality involved in the work-up of TED patients. It has shown superior activity in providing detailed soft-tissue assessment, when compared to computed tomography, and information regarding the activity status of the disease [9]. Active inflammation is typically associated with a hyperintense signal on STIR sequences of MRI, with greater water content being associated with higher signal intensity [4,10].

By assessing the MRI findings of 162 patients, we were able to identify specific structural MRI parameters which can aid the clinician in monitoring disease activity. Furthermore, we were also able to provide further evidence regarding well-established structural changes in the orbit associated with TED. 

In patients with active TED, exophthalmos showed a positive correlation with both orbital fat thickness (r = 0.55, p < 0.05) and total EOM volume (r = 0.344, p < 0.05). Similar correlations were observed in the quiescent group between exophthalmos and orbital fat thickness (r = 0.58, p < 0.05), as well as total EOM volume (r = 0.50, p < 0.05). The present findings are consistent with those of Liao and Huang [11], who identified a positive correlation between retro-bulbar volume reduction and the volume of orbital fat excised, thereby providing further support for the clinical utility of less invasive fat-removal decompressive techniques.

During this study, we were also able to assess the impact of orbital fat hypertrophy on the optic nerve. Patients with active TED were reported to have a statistically significant inverse correlation between optic nerve diameter and orbital fat thickness (Pearson’s r = -0.23, p = 0.049), whilst no correlation was observed in those with inactive TED (Pearson’s r = -0.06, p = 0.61). These findings suggest that patients with active TED are at a higher risk of developing optic neuropathy due to orbital fat hypertrophy. Hence, this group of patients might benefit from regular optic nerve testing, including, but not limited to, colour vision, contrast sensitivity, visual field testing, optic nerve optical coherence tomography, and visual evoked potentials [12].

Over the course of this analysis, the sensitivity and specificity of quantitative MRI-derived ratios were determined to assess disease activity associated with TED. The total EOM/exophthalmos ratio, with a cut-off value of 0.647 (AUC = 0.96; p < 0.05), and the IR/total EOM ratio, with a cut-off of 0.362 (AUC = 0.94; p < 0.05), demonstrated statistically significant diagnostic performance, supporting their utility in both the diagnosis and monitoring of disease activity in TED via MRI.

The orbital fat thickness/total EOM ratio, with a cut-off value of 0.66 (AUC = 0.91; p < 0.05), demonstrated a statistically significant ability to differentiate between active and inactive TED. Whilst its diagnostic accuracy is lower than that of the previously discussed ratios, its performance remains clinically meaningful, offering a valuable adjunctive marker for disease activity assessment in routine MRI evaluations.

In contrast, the IR/exophthalmos (AUC = 0.68; p < 0.05) and IR/orbital fat thickness (AUC = 0.61; p < 0.05) ratios demonstrated limited diagnostic performance, indicating inferior accuracy compared to the EOM-to-exophthalmos and total EOM ratios. Likewise, the orbital fat thickness/exophthalmos ratio (AUC = 0.58; p < 0.05) exhibited poor sensitivity and specificity, rendering it clinically unreliable. As such, these ratios lack sufficient diagnostic utility and should not be used to inform clinical decision-making in the assessment of TED. 

These findings are consistent with the established pathophysiology of TED, in which enlargement of the EOM during the active phase reflects inflammatory oedema and extracellular matrix expansion, including accumulation of glycosaminoglycans and collagen, with progression to fibrosis in chronic disease. Accordingly, the observed increases in EOM diameters, particularly involving the IR and MR muscles, should be interpreted as manifestations of inflammatory and remodelling processes rather than increased neural muscle tone or true contractile hypertrophy. This distinction has important clinical implications, as anti-inflammatory therapies are primarily effective in the active inflammatory phase, whereas fibrotic restrictive disease is generally managed surgically once disease inactivity is established.

Being a retrospective study, and with a predefined set of criteria, we were able to reduce to a minimum the selection bias associated with the recruitment of eligible participants. Furthermore, intervariability related to the measurements of MRI parameters was also reduced by utilising the same individuals for imaging assessment. 

Nevertheless, this study has several limitations. First, as a single-centre investigation, the study lacked sufficient comparative data, underscoring the need for external validation. Future research should ideally adopt a multicentre, prospective design to further minimise observational bias. Second, our analysis did not include other orbital structures affected in TED, most notably the lacrimal glands. The latter’s involvement in TED is well-documented [13,14], and data collection in this study might have provided further insight into the monitoring of TED. Finally, despite implementing multiple measures to reduce inter-observer variability, residual measurement differences may still have influenced the results. Future studies incorporating larger patient cohorts and a broader assessment of orbital structures are warranted to enhance the reproducibility and generalizability of findings.

## Conclusions

Our findings suggest that quantitative MRI-derived measurements, particularly total EOM volume and IR dimensions, may be associated with disease activity in TED. Ratios such as total EOM/exophthalmos and IR/total EOM demonstrated promising sensitivity and specificity in distinguishing active from inactive disease, indicating potential utility as objective imaging biomarkers. Whilst these results are encouraging, they are preliminary and hypothesis-generating; prospective, multicentre studies incorporating additional orbital structures and automated measurement techniques are warranted to validate these metrics and determine their clinical applicability in guiding management and monitoring disease progression in TED.
