# Correction: Quantitative Magnetic Resonance Imaging Biomarkers in the Evaluation of Disease Activity in Thyroid Eye Disease: A Retrospective Study

**DOI:** 10.7759/cureus.c410

**Published:** 2026-03-06

**Authors:** Duncan Marston, Sarah Grech, Andrea Noah Paris, Nicole Galdes, Isaac Bertuello, Andrew Palmier

**Affiliations:** 1 Ophthalmology, Mater Dei Hospital, Msida, MLT; 2 Surgery, Mater Dei Hospital, Msida, MLT

This article has been corrected as follows. The titles and legends of Figures 1 and 2 have been revised to indicate that Figure 1 is showing a T2-weighted image and Figure 2 is showing a T1-weighted image.

